# Genetic characteristics and epidemiology of inherited retinal degeneration in Taiwan

**DOI:** 10.1038/s41525-021-00180-1

**Published:** 2021-02-19

**Authors:** Ta-Ching Chen, Ding-Siang Huang, Chao-Wen Lin, Chang-Hao Yang, Chung-May Yang, Victoria Y. Wang, Jou-Wei Lin, Allen Chilun Luo, Fung-Rong Hu, Pei-Lung Chen

**Affiliations:** 1 Department of Ophthalmology, National Taiwan University Hospital, Taipei, Taiwan; 2 Graduate Institute of Clinical Medicine, College of Medicine, National Taiwan University, Taipei, Taiwan; 3 Department of Ophthalmology, College of Medicine, National Taiwan University, Taipei, Taiwan; 4 Case Western Reserve University School of Medicine, Cleveland, OH USA; 5 Department of Internal Medicine, National Taiwan University Hospital Yunlin Branch, Yunlin County, Taiwan; 6 Graduate Institute of Medical Genomics and Proteomics, College of Medicine, National Taiwan University, Taipei, Taiwan; 7 Department of Medical Genetics, National Taiwan University Hospital, Taipei, Taiwan

**Keywords:** Disease genetics, Genetic testing

## Abstract

Inherited retinal degenerations (IRDs) are a group of phenotypically and genotypically heterogeneous disorders with substantial socioeconomic impact. In this cohort study, we tried to address the genetic characteristics and epidemiology of IRDs in Taiwan. Totally, 312 families with IRDs were identified and recruited and genetic testing was performed via probe capture-based NGS targeting 212 IRD-related genes. Statistical analysis was based on the proband of each affected family. Disease-causing genotypes were identified in 178 families (57.1%). *ABCA4* variants were the most common cause of disease in this cohort (27 families, 15.2%), whereas *CYP4V2* variants were the most common cause for the single phenotype—Bietti’s crystalline dystrophy (12 families, 3.8%). Some variants such as *ABCA4*:c.1804C>T, *CYP4V2*:c.802-8_810delinsGC, and *EYS*:c6416G>A were population-specific disease-causing hotspots. Probands affected by *ABCA4*, *RPGR*, *RP1L1*, and *CEP290* sought medical help earlier while patients affected by *EYS* and *CYP4V2* visited our clinic at an older age. To evaluate the representativeness of our cohort in the genetic epidemiology of IRDs in Taiwan, our demographic data were compared with that of the total IRD population in Taiwan, obtained from the National Health Insurance Research Database. This is currently the largest-scale, comprehensive study investigating the genetic characteristics and epidemiology of IRD in Taiwan. These data could help patients and caregivers to adopt precision genomic medicine and novel gene therapies in near future.

## Introduction

Inherited retinal degenerations (IRDs) are a group of phenotypically and genotypically heterogeneous disorders with variable penetrance and severity. The prevalence of monogenic IRDs is approximately 1 in 2000 individuals, affecting more than two million people worldwide^1^. There are more than 20 IRD phenotypes, including rod-dominated diseases, cone-dominated diseases, generalized retinal degenerations, and vitreoretinopathies. Irreversible progression toward blindness due to IRD significantly affects quality of life. Severe visual impairment leads to reduced mobility and independence, posing heavy psychological and economic burden^2^.

However, diagnosing IRD is challenging due to its genotypic and phenotypic heterogeneity. Furthermore, many IRDs have overlapping clinical features. For example, retinitis pigmentosa (RP), the most common form of IRD, demonstrates primary rod cell dysfunction followed by cone cell degeneration and the clinical presentation may overlap with that of Leber congenital amaurosis (LCA) and even cone-rod dystrophy (CRD)^3–5^. Conversely, the same gene can encode different phenotypes. For example, IRDs with underlying *ABCA4* mutations have been associated with inherited macular degeneration, fundus flavimaculatus, generalized choriocapillaris dystrophy, and rapid-onset chorioretinopathy^6–10^. To further complicate diagnosis, many IRDs present similarly in late stages, with features such as severe retinal cell death, extensive atrophy of the retina, and irreversible visual loss.

In the era of precision medicine and gene therapy, genetic diagnosis for patients with IRD has become increasingly important. Up to 271 genes listed on RetNet (https://sph.uth.edu/retnet/) have been associated with IRDs. These genes have varying inheritance patterns and encode a wide spectrum of proteins, including structural and transmembrane proteins, proteins for phototransduction, and visual cycle proteins^11^. Identifying the disease genotype of IRDs is thus important for subsequent therapeutic strategies.

Previously, patients with IRD were underdiagnosed because of genotypic/phenotypic heterogeneity and the high cost of genetic diagnosis^12^. Recently, next-generation sequencing (NGS) technologies have been applied for genetic diagnosis, allowing timely and cost-effective detection of novel or rare variants in patients with IRDs; NGS has thus become the mainstream technique for refining diagnosis of IRDs by exploring the disease-causing genes. Large-scale genetic screening studies for IRDs have been previously performed in several countries^13–20^. However, a similar nationwide investigation for IRDs has not been done in Taiwan.

Taiwan is an isolated and highly developed island in East Asia, and is one of the most densely populated areas worldwide. Ethnically, Taiwan is relatively homogeneous; the population is composed of indigenous Taiwanese Austronesians and Han Taiwanese that immigrated from continental East Asia^21^. Taiwan is geographically isolated, and is relatively homogenous genetically. To explore the nationwide epidemiology and genetic aspects of IRDs in Taiwan, our team, granted by National Taiwan University Hospital, started performing genomic surveillance for IRD patients using a capture-based NGS platform in 2015. In addition to providing clinical and molecular surveillance for our own patients, we also accepted patient referrals from other medical centers in Taiwan. In this project, the Taiwan inherited retinal degeneration project (TIP), we aim to report the detailed clinical and genetic characteristics of IRD patients in Taiwan. In this study, we present the TIP database with respect to the molecular characteristics and genetic epidemiology of IRD and highlight the representativeness of the entire IRD-affected population in Taiwan based on statistical data from the national health insurance.

## Results

### Phenotypic characteristics

The average age at recruitment of the probands with IRD from 312 families was 43.2 years (range 6 months to 83 years old). One hundred and sixty-three probands were female (52.2%). Disease-causing variants were identified through panel-based NGS testing in 178 probands with a diagnostic rate of 57.1%. The clinical diagnosis of every proband was evaluated collectively by genotype and phenotype. Approximately two-thirds of the patients were diagnosed with retinitis pigmentosa (RP, *n* = 205, 65.7%), the most prevalent phenotype in our cohort, followed by macular dystrophy (MD, *n* = 39, 12.5%), cone dystrophy/cone-rod dystrophy (CD/CRD, *n* = 19, 6.1%), Leber congenital amaurosis (LCA, *n* = 16, 5.1%), Bietti’s crystalline dystrophy (BCD, *n* = 12, 3.8%), occult macular dystrophy (OMD, *n* = 5, 1.6%), retinoschisis (RS, *n* = 4, 1.3%), choroideremia (*n* = 2, 0.6%), familial exudative vitreoretinopathy (FEVR, *n* = 2, 0.6%), Alstrom syndrome (*n* = 2, 0.6%), and others (*n* = 6, 1.9%) (Table 1 and Supplementary Data 1).

**Table 1:** Demographic Data of TIP Cohort.

Phenotype	No. of proband (%)	Age at examination (yr, mean ± SD)	Female sex	Onset age (yr, mean ± SD)	Patient with positive FH	Patient with negative FH
RP	206 (66.0%)	48.22 ± 15.15	108 (52.4%)	30.65 ± 17.61	66	140
MD	39 (12.5%)	40.91 ± 20.66	25 (64.1%)	29.42 ± 19.25	9	30
CD/CRD	19 (6.1%)	41.18 ± 14.64	7 (36.8%)	3 ± 15.89	2	17
LCA	16 (5.1%)	16.10 ± 13.10	5 (31.3%)	3.75 ± 2.79	2	14
BCD	11 (3.5%)	48.13 ± 11.75	10 (90.9%)	36.64 ± 13.41	5	6
OMD	5 (1.6%)	29.84 ± 15.57	3 (60.0%)	18.75 ± 7.89	2	3
RS	4 (1.3%)	19.35 ± 18.57	0	4.00 ± 3.46	3	1
Choroideremia	2 (0.6%)	35.01 ± 15.38	0	4.50 ± 4.95	2	0
FEVR	2 (0.6%)	2.05 ± 1.80	0	0.89 ± 0.16	1	1
Alström syndrome	2 (0.6%)	4.83 ± 1.28	1 (50.0%)	1.50 ± 0.71	1	1
others^a^	6 (1.9%)	48.66 ± 20.16	4 (66.7%)	22.83 ± 21.02	2	4
Total	312	43.91 ± 18.15	163 (52.2%)	28.17 ± 18.43	95	217

*FH* family history.

^a^Others indicated that the phenotype is less than two probands or unclear phenotype in our cohort, including Congenital Stationary Night Blindness, vitreoretinochoroidopathy, Sorsby fundus dystrophy, and Oguchi disease.

### Inheritance patterns and family history

**Fig. 1:**
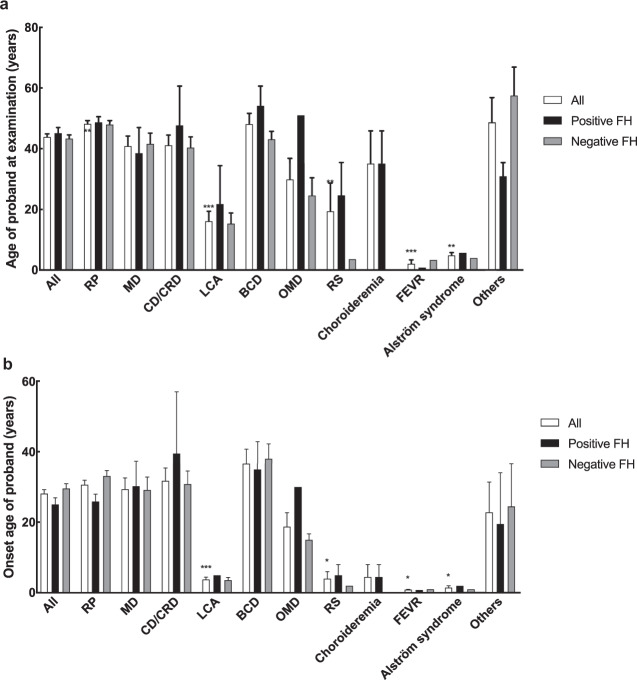
The age at examination and at onset of IRD probands by phenotype. **a** The age at examination of all probands or of probands with a positive/negative family history. **b** The onset age of all probands or of probands with a positive/negative family. Means with different symbols are statistically significant compared to all probands by Fisher’s LSD test (***p < 0.001; **p < 0.01; *p < 0.05). FH family history.

Of all IRD cases, 217 (69.6%) were sporadic (i.e., patients with a negative family history of IRD) and 95 (30.4%) were inherited (i.e., patients with a positive family history of IRD). The number of RS and choroideremia probands with a positive family history (FH) was higher than that of patients with a negative FH (Table 1). The age at onset and at examination of the probands are shown in Fig. 1a, b, respectively. There were no significant differences between positive and negative FH in each clinical phenotype. However, on average, probands with LCA, RS, choroideremia, FEVR, and Alström syndrome tended to have an earlier age of onset and visited our clinic at a younger age, whereas patients with RP tended to visit our clinic at an older age (Fig. 1b). The distribution curve of the age at diagnosis was shown in Supplementary Fig. 1.

**Fig. 2:**
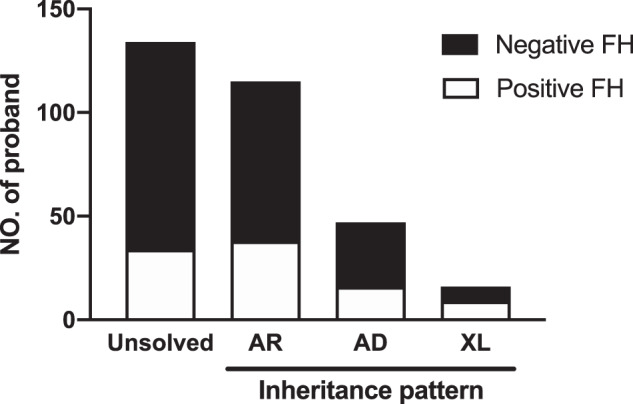
Classification of the inheritance patterns of probands with a positive/negative family history. FH family history, AR autosomal recessive inheritance, AD autosomal dominant inheritance, XL X-linked inheritance.

The inheritance patterns were primarily based on the genetic results and then we confirmed the pattern with pedigree for each family. For those cases without definite genetic diagnosis, the inheritance patterns were imputed from clinical observation. When classified according to inheritance pattern, 64.6% (115/178) harbored gene variants with autosomal recessive (AR) inheritance, whereas 26.4% (47/178) had autosomal dominant (AD) inheritance and 9.0% (16/178) had X-linked (XL) inheritance (Fig. 2). Among these three inheritance patterns, patients with XL inheritance had the highest chance of positive FH (56.3%, 9/16) compared to the AD (34.0%, 16/47) and AR (33.0%, 38/115) inheritance patterns.

### Genetic characteristics

**Fig. 3:**
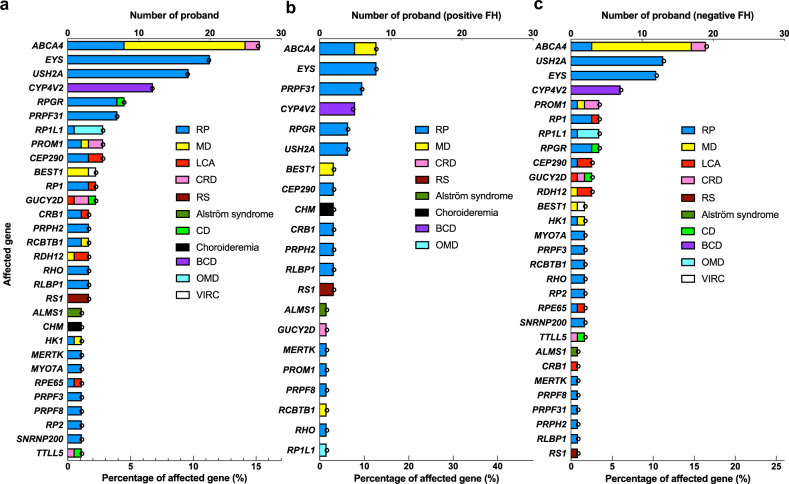
Number of probands grouped by disease-causing gene, phenotype, and the percentage of disease-causing gene in the probands in our cohort shown by a hollow circle. **a** Number of probands by disease-causing gene and phenotype, and the percentage of disease-causing genes in 312 probands. Only the disease-causing genes in two or more probands are shown. **b** Number of probands by disease-causing gene and phenotype and the percentage of disease-causing genes in probands with a positive family history. **c** Number of probands by disease-causing gene and the phenotype and percentage of disease-causing genes in probands with a negative family history. FH family history, RP retinitis pigmentosa, MD macular dystrophy, LCA Leber congenital amaurosis, CRD cone-rod dystrophy, RS retinoschisis, CD cone dystrophy, BCD Bietti’s crystalline dystrophy, OMD occult macular dystrophy, VIRC vitreoretinochoroidopathy.

To identify the disease-causing variants in patients with IRD, 212 IRD-related genes were sequenced by capture-based NGS technology. Disease-causing variants were identified in 178 probands, corresponding to a detection rate of 57.1% (178/312). Figure 3a lists all the disease-causing genes influencing at least two or more probands in our cohort. Among the analyzed genes, biallelic variants of *ABCA4* were the most frequent disease-causing variants (27/178, 15.2%), responsible for diseases in 27 families, including 17, 8, and 2 families with STGD, RP, and CRD, respectively. Other genes responsible for more than 2% of the probands in our cohort included *EYS, USH2A, CYP4V2, RPGR, PRPF31, RP1L1, PROM1, CEP290, BEST1, RP1*, and *GUCY2D*. These 12 genes were responsible for 118 families (118/178, 66.3% of the genetically diagnosed probands) in our cohort. The disease-causing genes of probands with positive or negative FH were analyzed further (Fig. 3b, c). Among them, probands with *PRPF31* (6/7, 86%), *RPGR* (4/8, 50%), *CHM* (2/2, 100%), *CRB1* (2/3, 67%), *PRPH2* (2/3, 67%), *RLBP1* (2/3, 67%), *RS1* (2/3, 67%), and *BEST1* (2/4, 50%) mutations tended to have a known family history. Except *CRB1* and *RLBP1*, most of these genes followed an AD or XL inheritance pattern. Conversely, probands diagnosed with *ABCA4, USH2A, RP1L1, PROM1, PR1, GUCY2D, RDH12, HK1, MYO7A, RPE65, PRPF3, RP2, SNRNP200*, or *TTLL5* mutations had mostly negative FH (>70% of proband in each gene).

### Novel and common disease-causing variants

We further distinguished the novel variants from previously reported pathogenic variants in disease-causing genes. In our cohort, 68 novel variants were identified from 76 probands, including deletion, insertion, duplication, and noncoding variants (Supplementary Table 1). Among the genes analyzed, *USH2A*, identified in nine RP probands, was responsible for the most novel variants in our cohort (9/76, 11.8%), followed by *EYS* (6/76, 7.9%), *ABCA4* (5/76, 6.6%) and. *CEP290* (5/76, 6.6%). In probands with positive FH. *PRPF31* and *RPGR*, which follow AD and XL inheritance patterns, respectively, were the major disease-causing genes with novel variants, and were responsible for 3 and 4 RP families, respectively. On the contrary, *USH2A* was responsible for most novel variants in probands with negative FH, and was identified in eight probands with RP. Supplementary Table 2 lists the high-frequency variants identified in our cohort. We further evaluated the variants of affected genes enriched in our cohort by comparing them with public datasets, including the Taiwan Biobank and East Asian population data in The Genome Aggregation Database (gnomAD). Several variants in our present cohort are disease-causing hotspots, including *ABCA4:*c.1804C>T*, CYP4V2:*c.802-8_810delinsGC, and *EYS*:c6416G>A, which were identified in more than ten probands in our cohort.

### Relationship between genotype and age of onset

**Fig. 4:**
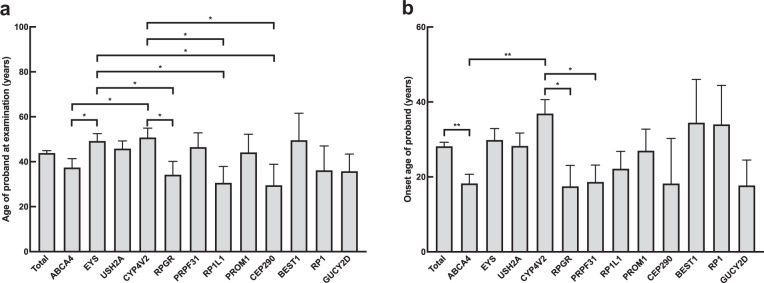
The age at examination and age at onset of IRD probands grouped by the disease-causing gene. **a** The age at examination of all probands. **b** The onset age of all probands. Mean with different symbols are statistically significant compared to all probands by Fisher’s LSD test (**p < 0.01; *p < 0.05).

Among the 12 disease-causing genes each affecting more than 3% of our cohort, patients affected by *ABCA4, RPGR, RP1L1*, and *CEP290* sought medical help at an earlier age, whereas patients affected by *EYS* and *CYP4V2* visited our clinic at an older age (Fig. 4a). Further, patients affected by *ABCA4, RPGR*, and *PRPF31* had an earlier age of onset, whereas patients affected by *CYP4V2* had a relatively later age of onset (Fig. 4b).

### Representativeness for total IRD population in Taiwan

**Fig. 5:**
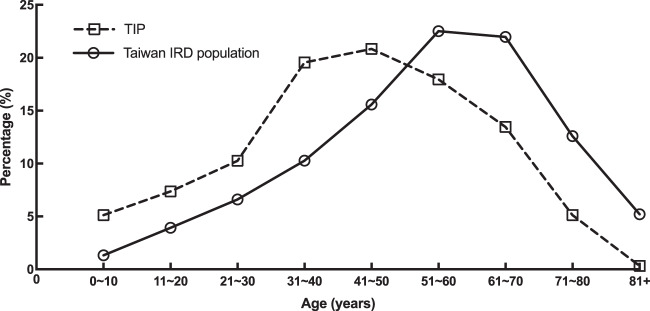
Age distribution of TIP and Taiwan population. Age distribution of our cohort (TIP) and the Taiwan IRD population based on the National Health Insurance Research Database (NHIRD) of Taiwan.

To evaluate the representativeness of our cohort in the genetic epidemiology of IRDs in Taiwan, we searched the National Health Insurance Research Database (NHIRD) of Taiwan for the current number of patients diagnosed with hereditary retinal disorders (ICD-10-CM codes, H31101, H3550, H3552, H3553, and H3554) and their age distribution. In all, 6674 patients with ICD-10-CM codes were identified from the Taiwan NHIRD, indicating that our present cohort (312 families and 587 patients approached) accounted for nearly 9% of all IRD patients in Taiwan. A comparison of age distribution between our cohort and Taiwan’s IRD population as a whole, based on the NHIRD, is shown in Fig. 5. The age distribution of our cohort showed a similar trend compared to the total IRD population in Taiwan. However, in general, our patients seemed to be a little younger than the IRD population overall. In reviewing the pedigrees in our cohort, we noticed that within an affected family, younger members were more motivated to seek medical advice.

## Discussion

IRD is an important cause of visual impairment, especially in working age adults, thus significantly impacting the quality of life of both patients and their families. IRD can be inherited in autosomal dominant, autosomal recessive, or X-linked patterns. However, rare mitochondrial and digenic forms of retinal dystrophies have also been characterized. Currently, more than 270 causative genes have been identified for IRD, and more are continuously being explored^22–25^. Panel-based NGS or WES can be effective tools for detecting IRD-causing mutations. In recent years, a variety of NGS studies have employed target panel-based NGS approaches to sequence the exons of known IRD genes for their patients^18,26–28^. These studies usually report a success rate of 50–60% in identifying the disease-causing mutations in patients with IRD. To increase the ability in detecting disease-causing variants, DNA fragments were generated with a peak length of 800 bp in library preparation. The main advantage of longer DNA fragments was that longer reads may align to the DNA regions which contained repetitive sequences by performing paired-end sequencing, such as ORF15 region of *RPGR*. In addition, we captured the entire genomic sequence of common disease-causing genes, such as *ABCA4*, *CEP290*, *USH2A*, and annotated the intronic variants by SpliceAI algorithm^29^.

In this study, we present our cohort (patients recruited during July 2015 and June 2019) of the Taiwan Inherited retinal degeneration Project (TIP), based on patients visiting National Taiwan University Hospital, a tertiary medical center. To maximize this project’s representativeness of the real-world distribution of IRD in Taiwan, we recruited all patients referred to us with a clinical diagnosis of IRD who agreed to genetic testing. In this 48-month period, a total of 312 families and 587 patients with IRD were approached. To eliminate the quantitative bias from multiple affected members within a single family, only the 312 probands (the first affected patient of a given family to visit our clinic) were included for statistical analysis in this study. Our results revealed a diagnostic rate of 57.1% using a capture-based NGS approach targeting 212 IRD-related genes. This diagnostic rate is similar to previous reports from other countries, and may be further enhanced by whole exome or whole genome sequencing in future studies. To the best of our knowledge, this is the first large-scale, comprehensive study characterizing the genetic aspects and epidemiology of IRD in Taiwan.

Taiwan is a continental island in East Asia and has a relatively homogeneous population. Taiwan was periodically connected to mainland Asia during the Pleistocene glacial period^30^; consequently, the Taiwanese population shares many genetic variants with mainland Asian populations. Furthermore, Taiwan also has close contact with neighboring countries, such as Japan, Southeast Asian countries, and Oceania countries. Comparing the distribution of disease-causing genes in our cohort to those of data from similar studies in China and the US^13,20^, we found that all three cohorts shared some common disease-causing genes such as *EYS, USH2A, ABCA4, RPGR, PRPF31*, and *CEP290*. Some genes, such as *PROM1* associated with RP, CRD, and Stargardt disease, as well as *CYP4V2* associated with Bietti crystalline dystrophy, were frequently found in our cohort and the cohort from China but were less frequent in the US cohort. In our cohort, disease-causing *PROM1* variants were found in five families (2.8% of all solved families) with diverse RP, CRD, and MD phenotypes. *CYP4V2* variants were responsible for an even larger portion of our cohort (12 families, 6.7% of solved families); all affected patients had Bietti crystalline dystrophy, exclusively. Conversely, some genes such as *RHO, PRPH2*, and *CRB1* that were rare in our cohort were frequently seen in the US cohort.

Notably, *ABCA4*, which was responsible for 27 families (15.2% of solved families) in our cohort, was the single most common disease-causing gene in the cohort, far exceeding other genes in frequency. This phenomenon mirrors the data from the US cohort where *ABCA4* was responsible for 17.3% of the cases^20^. However, the prevalence of *ABCA4* was not as high in a Mainland China cohort study published by Huang et al.^13^. *ABCA4* variants are known to be a major cause of Stargardt disease as well as some autosomal recessive RP. However, macular dystrophy, including Stargardt disease, accounts for only 12.5% (39 of 312 probands) of our cohort, which is much lower than 28.2% in the US cohort. Furthermore, there were 3 disease-causing hotspots, each detected in more than 10 probands of our cohort, including *EYS*:c.6416G>A (p.Cys2139Tyr), *CYP4V2*:c.802-8_810delinsGC, and *ABCA4*:c1804C>T (p.Arg602Trp). In prior studies, *ABCA4*:c1804C>T (p.Arg602Trp) was seldom reported as a common variant^10,31^. As Taiwan is a relatively isolated island, in future studies, it may be worth investigating whether a founder effect exists among these families, which could account for high prevalence of this variant among our population. This is significant because novel therapies emphasize the precise diagnosis of genetic variants.

IRD prevalence is believed to have been underestimated in the past because a lack of effective treatment deterred patients from seeking medical consultation. However, with the development of new techniques for molecular diagnosis and novel therapies such as AAV-mediated gene augmentation in recent years, the situation has changed^32,33^. In our study, comparison of patients with known versus unknown family histories showed no difference in when they first became aware of visual disturbances and their timing of seeking medical advice (Fig. 1). However, patients with certain phenotypes such as LCA, RS, FEVR, and Alström syndrome, obviously experienced symptoms earlier and thus visited the clinic earlier. Notably, among the common disease-causing genes (>3% of our solved families), probands with *EYS* and *CYP4V2* variants sought medical attention at an older age, whereas probands with *ABCA4*, *RPGR, RP1L1*, and *CEP290* did so at a significantly younger age. When recalling their age of symptom onset, *CYP4V2* probands had a significantly later onset compared to *ABCA4*, *RPGR*, and *PRPF31* probands. This is reasonable when we correlate the clinical phenotypes; *CEP290* is related to LCA, *RP1L1* is related to occult macular dystrophy (OMD), *ABCA4* is related to Stargardt disease and severe forms of autosomal recessive RP, and *CYP4V2* is related to BCD. However, this may also indicate that RP with different genotypes may have different courses of progression. Some studies have discussed the different prognoses of individual disease-causing genes for RP; patients with *PRPH2* mutations had a relatively good prognosis^34^, whereas patients with *PRPF31* mutations had early macular involvement and central vision loss, which is important for daily life^35^. We also found that there was different prevalence of the disease-causing genes between patients with and without family history. Despite some popular disease-causing genes that ranked high in both groups, some genes may have quite different prevalence between the two groups. For example, *PRPF31* is the third popular disease-causing gene in patients with positive family history while ranked 26^th^ in patients without known family history. The uneven distribution might provide scientific clues for future studies such as exploring the probability of de novo mutations for individual genes.

We initiated this study—the Taiwan Inherited retinal degeneration Project (TIP)—to recruit IRD patients from multiple medical centers in different areas of Taiwan. All patients were clinically and molecularly diagnosed at a single referral medical center, the Department of Ophthalmology, National Taiwan University Hospital and Graduate Institute of Medical Genomics and Proteomics, National Taiwan University, to eliminate possible deviation among different settings and retinal specialists. In this report of the TIP, we aimed to standardize the data-analysis pipeline and to characterize the diversity of mutations in patients with IRD in our cohort. To confirm the cohort’s representativeness of IRD in Taiwan’s population as a whole, we also referred to the population statistics from Taiwan’s National Health Insurance Research Database (NHIRD), which has records of medical visits and ICD codes for every resident in Taiwan. The statistical results showed that the patients included in our current cohort account for 8.8% of currently diagnosed IRD patients in Taiwan. Figure 5 further confirms that the age distribution of our cohort follows a similar trend as that of the total population with IRD in Taiwan with a small left shift, indicating that our probands were slightly younger than the general IRD population on average.

Our study has some limitations. First, the panel-based genetic test predominantly targeted exons of 212 genes known to cause IRD. It is possible that some currently undiagnosed families have genetic variations in uncovered areas of these genes or variations in novel uncovered genes. Secondly, information regarding pedigrees and symptom onset largely depended on the patients’ memories and may thus be erroneous, even though we tried our best to access more family members for accuracy. Third, because IRDs are rare disorders, it is statistically challenging to analyze the different prognosis among different genotypes within the same population due to the limited number of probands for each genotype in Taiwan. Further analysis may be possible in the future, as the project proceeds and more families are registered and analyzed. Fourth, the judgement of family history could be imperfect. For example, in our cohort, a significant majority of the probands in autosomal dominant IRD group did not have family history by their self-report. This may due to that the nuclear family style has become the mainstream in current Taiwan society. The probands could be the only one kid in his/her generation without brothers and sisters while the parents may be underdiagnosed due to the lack of medical resource before. In some cases, the incomplete penetrance of IRD-related genes may also play a role to make the judgement even more difficult. Finally, some patients of IRD could be underdiagnosed. Therefore, the total number calculated via NHIRD may be underestimated compared to the reality. Despite these limitations, we believe that the present results are valuable. This is a large-scale study on the genetic characteristics and epidemiology of IRD in Taiwan. Furthermore, as regional differences are an important factor when considering genetic diseases, a better understanding of the genetic distribution of IRD in Taiwan is crucial to incorporate novel gene therapy in the near future.

In summary, this report of the Taiwan Inherited retinal Degeneration Project presents the phenotype classification, genetic characteristics, and related statistical analysis of 312 families with IRD. With these data, we hope to achieve better understanding and care for patients with IRD, as well as provide comparative data for IRD specialists worldwide. To the best of our knowledge, this is the first large-scale, comprehensive study investigating the genetic characteristics and epidemiology of IRD in Taiwan.

## Methods

### Subjects

The present study was conducted at the Department of Ophthalmology, National Taiwan University Hospital (NTUH), and was approved by the Research Ethics Committee of the National Taiwan University Hospital (IRB NO.: 201408082RINC). Patients with IRD were recruited either from our out-patient clinic or from patients referred by other medical centers in Taiwan to our hospital (please see Acknowledgements for the list of medical centers).

From July 2015 to June 2019, 312 families with IRDs were identified and recruited consecutively into our TIP project. During a 48-month period, 587 patients of IRDs in the 312 families were approached and the first patient of each family recruited into our project was regarded as the “proband.” This statistical analysis in this study is based on the proband of each affected family to minimize the potential bias in genetic epidemiology. For every subject recruited, detailed ophthalmological examinations were performed, including measurement of best-corrected visual acuity, electroretinograms, color fundus photography, optical coherence tomography, and fundus autofluorescence imaging to confirm the clinical diagnosis of IRDs. A detailed medical history, including pedigree, was obtained for every proband. Blood samples were collected in EDTA tubes after obtaining informed consent, and genomic DNA was extracted from peripheral blood mononuclear cells using a DNA extraction kit (Gentra Puregene Blood Kit, QIAGEN, Hilden, Mettmann, Germany).

### Capture-based target enrichment followed by NGS

Using the genomic DNA, we performed genetic testing via a probe capture-based NGS approach targeting 212 IRD-related genes (Supplementary Table 3). The test results, along with family history and clinical diagnosis, were used to evaluate the genotype and phenotype of IRDs. DNA fragment libraries were generated by sonication (Covaris, Woburn, MA, US). Fragmented gDNAs, with a peak length of 800 bp, were tested for size distribution and concentration using the Agilent Bioanalyzer 2100 (Agilent Technologies, Santa Clara, CA, US) and Qubit (Thermo Scientific, Waltham, MA, US). Libraries were generated using the TruSeq Library Preparation Kit (Illumina, San Diego, CA, US). Probe-based target enrichment was then performed to target the transcripts of all 212 genes implicated in retinal degeneration using the SeqCap EZ Hybridization and Wash Kit (Roche NimbleGen, Madison, WI, US). These 212 genes were selected from the RetNet database (https://sph.uth.edu/retnet/), OMIM database (https://www.ncbi.nlm.nih.gov/omim), and peer-reviewed publications (PubMed search queries: hereditary retinal dystrophy). All exons (including the 5′ and 3′ untranslated regions) with at least 100 bp flanking intron sequences of the 212 genes were defined as targeted regions, which summed up as a 3.6 Mbp region (Table S3). In addition to capturing exons, we captured the entire genomic sequence, including both exons and introns for some genes (*USH2A, OFD1, ABCA4, PRPF31 CEP290, RPGR, GUCY2D, KCNV2, CNGB3, CNGA3, PRPF4, MYO7A, RPGRIP1, RDH12, AIPL1, CNGB1, NRL, SPATA7, FAM161A, RPE65, PDE6A, PDE6B, BBS10*, and *BBS1*) to increase the ability of detecting structural variants and possible intronic disease-causing variants. Paired-end sequencing was achieved using Illumina MiSeq or NextSeq 550 (Illumina, Inc. San Diego, CA, US). The capture probes of 212 IRD-related genes were designed using NimbleDesign (https://design.nimblegen.com/) and produced by Roche NimbleGen (Roche NimbleGen, Madison, WI, US). The mapped reads (GRCh37/hg19) reached a mean coverage depth of 135.4-fold, and 95.4% of targeted regions were covered by 20 or more reads.

### Data analysis

The paired-end reads were analyzed for mapping to the human reference genome (Feb. 2009 GRCh37/hg19) using BWA-MEM (version 0.7.12). Picard (version 1.54) was then used to perform data conversion, sorting, and indexing. Variant calling for single-nucleotide variants (SNVs) and small insertions/deletions (INDELs) was conducted using GATK package software (version 3.4). ANNOVAR 2016Feb01 was used to annotate the allele frequency of variants based on ClinVar (https://www.ncbi.nlm.nih.gov/clinvar/), Genome Aggregation Database (gnomAD, https://gnomad.broadinstitute.org), NHLBI-ESP 6500 exome project (http://evs.gs.washington.edu/EVS/), 1000 Genomes project (http://www.1000genomes.org/), Exome Aggregation Consortium projects (ExAC, http://exac.broadinstitute.org/), 69 Genomes Data (CG69, http://www.completegenomics.com/public-data/69-genomes/), Kaviar Genomic Variant Database (http://db.systemsbiology.net/kaviar/), dbSNP Build 147 (avsnp147, https://www.ncbi.nlm.nih.gov/snp), and Taiwan Biobank (https://taiwanview.twbiobank.org.tw/index), which contains 1517 community-based healthy subjects; pathogenicity prediction was performed using SIFT, PolyPhen-2 HDIV, PolyPhen-2 HVAR, LRT, MutationTaster, FATHMM, MutationAssessor, PROVEAN, VEST, MetaSVM, MetaLR, MCAP, CADD, and DANN. The intronic variants were analyzed with SpliceAI algorithm^29^, which is implemented on TAIGenomics, a genomic analysis cloud (https://taigenomics.tw). The integrative genomics viewer (IGV) was then used to visualize the sequence mapping.

### Variant filtering

The data analysis pipeline is shown in the Supplementary Fig. 2. Briefly, after variant annotation, filtering was applied to decrease the number of false positive results by removing synonymous variants and variants with allele frequencies higher than 5% in either one of the population databases. Variants were comprehensively interpreted by Varsome which followed the American College of Medical Genetics and Genomics (ACMG) guidelines^36,37^. Every variant identified in our cohort was searched with literature search engines—Mastermind (https://www.genomenon.com/mastermind/) and variant2literature (https://variant2literature.taigenomics.com) for previous literature. Sanger sequencing was used to confirm the nucleotide change of variants that met the above criteria.

### Comparison with the epidemiology data of the total population

The epidemiology database of all IRD patients in Taiwan was obtained from Taiwan’s National Health Insurance Research Database (NHIRD), which is maintained by the National Health Research Institutes of Taiwan. The NHIRD data extracted for the present study included inpatient, outpatient, and pharmaceutical claims, and disease diagnoses coded according to the International Classification of Diseases, 10th Revision (ICD-10-CM). Data of patients diagnosed with hereditary retinal disorder (ICD-10-CM codes, H31101, H3550, H3552, H3553, and H3554) between January 2016 and December 2017 were retrieved. In all, 6674 patients with these ICD-10-CM codes were identified from the Taiwan NHIRD.

### Statistical analysis

The results in this study are shown as mean ± standard error of mean. Student’s *t*-test and analysis of variance (ANOVA) followed by post hoc multiple comparisons were performed using SPSS 12.0 (SPSS Inc., Chicago, IL, USA).

### Reporting summary

Further information on research design is available in the Nature Research Reporting Summary linked to this article.

## Supplementary information

Supplementary Information

Supplementary Data 1

Reporting Summary

## Data Availability

The sequencing raw data (FASTQ files) analyzed during the current study available in the NCBI Sequence Read Archive (SRA) (PRJNA681323). The data of non-sequencing data and materials are available on reasonable request from the corresponding authors.
