# Supplementary material for: Genetic characteristics and epidemiology of inherited retinal degeneration in Taiwan
Source: NPJ Genom Med. 2021 Feb 19;6:16. doi: 10.1038/s41525-021-00180-1 (PMC7896090; doi:10.1038/s41525-021-00180-1)
Supplement: Supplementary file 1 — Supplementary Information [file 41525_2021_180_MOESM1_ESM.pdf]

Genetic Characteristics and Epidemiology of Inherited Retinal Degeneration in Taiwan

Ta-Ching Chen[1,2], Ding-Siang Huang[1], Chao-Wen Lin[1], Chang-Hao Yang[1,3], Chung-May Yang[1,3], Victoria Y. Wang[4], Jou-Wei Lin[5], Allen Chilun Luo[6], Fung-Rong Hu[1,3*], Pei-Lung Chen[2,6,7*]

Corresponding authors:
Fung-Rong Hu, M.D.
E-mail: fungronghu@ntu.edu.tw

Pei-Lung Chen, M.D., Ph.D.
E-mail: paylong@ntu.edu.tw

Supplementary information
Contents

Supplementary Table 1 1
Supplementary Table 2 9
Supplementary Table 3 11
Supplementary Figure 1 13
Supplementary Figure 2 14

Additional Supplementary Data uploaded as a separate excel sheet:
Supplementary Data 1: The list of disease-causing variants for each proband in our cohort

Supplementary Table 1 Novel Variants Identified in The TIP Cohort

Gene variants	Allele count in TIP	Taiwan Biobank			GnomAD East Asia			*ACMG classification*
		Allele frequency	Odds ratio	*P*-value	Allele frequency	Odds ratio	*P*-value	
***ABCA4* (NM_000350.3)**								
c.3274G>T (p.Val1092Leu)	1	0.000991	1.6185	0.52755	0.000381	3.5568	0.26511	LP
c.4505G>A (p.Cys1502Tyr)	1	N.A.	Infinity	0.17059	N.A.	Infinity	0.030324	LP
c.4846_4847insT (p.Lys1616IlefsTer5)	1	N.A.	Infinity	0.17059	N.A.	Infinity	0.030324	P
c.5501T>C (p.Ile1834Thr)	1	0.00033	4.8652	0.31226	0.000869	1.7776	0.44311	LP
***ALMS1* (NM_015120.4)**								
c.5022C>G (p.Tyr1674Ter)	1	N.A.	Infinity	0.17059	N.A.	Infinity	0.030324	P
c.7528C>T (p.Arg2510Ter)	1	N.A.	Infinity	0.17059	N.A.	Infinity	0.030324	P
***BEST1* (NM_001139443.2)**								
c.34T>C (p.Tyr12His)	1	N.A.	Infinity	0.17059	N.A.	Infinity	0.030324	LP
c.35C>A (p.Ala12Asp)	1	N.A.	Infinity	0.17059	N.A.	Infinity	0.030324	LP
***CACNA1F* (NM_001256789.3)**								
c.3868_3869delAT	1	N.A.	Infinity	0.17059	N.A.	Infinity	0.030324	P

(p.Met1290AlafsTer8)								
***CEP290* (NM_025114.4)**								
c.6358-1G>A	2	0.000332	22.9847	0.018379	0.000601	5.3023	0.07199	P
c.6798G>A (p.Trp2266Ter)	5	0.002307	3.493	0.039531	0.00167	4.9202	0.005384	P
c.7333_7334insAGAAG (p.Val2445GlufsTer3)	1	N.A.	Infinity	0.17059	N.A.	Infinity	0.030324	P
***CHM* (NM_000390.4)**								
c.855dupA (p.Gln286ThrfsTer21)	1	N.A.	Infinity	0.17059	N.A.	Infinity	0.030324	P
***CNGB1* (NM_001297.5)**								
c.1536-41_1552del	1	N.A.	Infinity	0.17059	0.00002408	4.4753	0.22254	P
***CRB1* (NM_201253.3)**								
c.858T>A (p.Cys286Ter)	1	N.A.	Infinity	0.17059	N.A.	Infinity	0.030324	P
c.1564C>T (p.Leu522Phe)	1	N.A.	Infinity	0.17059	N.A.	Infinity	0.030324	LP
c.3957C>A (p.Phe1319Leu)	1	N.A.	Infinity	0.17059	N.A.	Infinity	0.030324	LP
***DRAM2* (NM_178454.5)**								
c.744delC (p.Asp248GlufsTer47)	1	0.000659	2.4334	0.42952	N.A.	Infinity	0.030324	LP

***EYS* (NM_001142800.2)**								
c.4365_4366insTTCT (p.Ser1456PhefsTer9)	1	N.A.	Infinity	0.17059	N.A.	Infinity	0.030324	LP
c.4991_4992insAAGA (p.Cys1665ArgfsTer19)	1	N.A.	Infinity	0.17059	N.A.	2.4992	0.49026	LP
c.5304_5314del (p.Asn1768LysfsTer2)	1	N.A.	Infinity	0.17059	N.A.	Infinity	0.030324	P
c.8309T>C (p.Leu2770Pro)	1	0.000330	4.8684	0.31211	0.000367	4.3724	0.243	VUS
c.8600delG (p.Gly2867ValfsTer5)	2	N.A.	Infinity	0.0063854	N.A.	Infinity	0.00091809	LP
c.9073G>A (p.Gly3025Arg)	1	N.A.	Infinity	0.17059	N.A.	Infinity	0.030324	VUS
c.9083_9084insA (p.Ser3029LeufsTer7)	1	N.A.	Infinity	0.17059	N.A.	Infinity	0.030324	LP
***GNAT1* (NM_000172.4)**								
c.863-2A>G	1	N.A.	Infinity	0.17059	N.A.	Infinity	0.030324	P
***GRK1* (NM_002929.3)**								
c.1338C>A (p.Cys446Ter)	1	0.000659	2.4334	0.42952	0.000565	2.7922	0.32947	P
***GUCY2D* (NM_000180.4)**								
c.2692_2695delCCCA	1	N.A.	Infinity	0.17059	N.A.	Infinity	0.030324	P

(p.Pro898LeufsTer29)								
c.*24+2T>G	1	0.001783	0.8986	1	0.000452	3.547	0.28076	LP
HK1 **(NM_000188.2)**								
c.1967A>G (p.Asn656Ser)	1	N.A.	Infinity	0.17059	N.A.	Infinity	0.030327	LP
IFT140 **(NM_014714.4)**								
c.1375T>G (p.Trp459Gly)	1	N.A.	Infinity	0.17059	N.A.	Infinity	0.030324	VUS
MERTK **(NM_006343.3)**								
c.1988_1989insAT (p.Ile664SerfsTer7)	1	N.A.	Infinity	0.17059	N.A.	Infinity	0.030324	P
NPHP1 **(ENST00000316534.4)**								
c.2158C>T (p.Gln720Ter)	1	N.A.	Infinity	0.17059	N.A.	Infinity	0.030324	P
PCDH15 **(NM_001142771.2)**								
c.4588_4589insGAGA (p.Asn1530ArgfsTer8)	1	N.A.	Infinity	0.17059	N.A.	Infinity	0.030324	LP
POC1B **(NM_001199777.2)**								
c.293dupT (p.Leu98PhefsTer24)	1	N.A.	Infinity	0.17059	0.000359	Infinity	0.030324	P

***PROM1* (NM_006017.3)**								
c.242dupA (p.Lys82GlufsTer4)	1	N.A.	Infinity	0.17059	N.A.	Infinity	0.030324	P
c.631-15_631-10delATGTTC	1	N.A.	Infinity	0.17059	N.A.	Infinity	0.030324	VUS
***PRPF3* (NM_004698.3)**								
c.-49+2T>C	2	N.A.	Infinity	0.0063854	0.000643	5	0.19877	LP
***PRPF31* (NM_015629.4)**								
c.1043A>C (p.Asp348Ala)	1	N.A.	Infinity	0.17059	N.A.	Infinity	0.030324	LP
c.1375-2A>G	1	N.A.	Infinity	0.17059	N.A.	Infinity	0.030324	P
***PRPH2* (NM_000322.5)**								
c.372_381delinsAAGCTGA (p.Leu126Ter)	1	N.A.	Infinity	0.17059	N.A.	Infinity	0.030324	P
c.536G>A (p.Trp179Ter)	1	N.A.	Infinity	0.17059	N.A.	Infinity	0.030324	P
***RHO* (NM_000539.3)**								
c.926T>A (p.Met309Lys)	1	N.A.	Infinity	0.17059	0.0000544	29.5201	0.064554	LP
***RP1* (NM_006269.2)**								
c.2290dupA (p.Thr764AsnfsTer17)	1	N.A.	Infinity	0.17059	N.A.	Infinity	0.030324	P

***RP1L1* (NM_178857.6)**								
c.485delC (p.Pro162LeufsTer32)	2	N.A.	Infinity	0.0063854	N.A.	Infinity	0.00091809	LP
***RP2* (NM_006915.3)**								
c.97G>T (p.Glu33Ter)	1	N.A.	Infinity	0.17059	N.A.	Infinity	0.030324	P
***RPE65* (NM_000329.3)**								
c.1154C>T (p.Thr385Met)	3	0.002966	1.6237	0.44206	0.00299	1.5242	0.45446	LP
***RPGR* (NM_001034853.2)**								
c.614dupT (p.Thr206AsnfsTer5)	1	N.A.	Infinity	0.17059	N.A.	Infinity	0.030324	P
c.1991C>G (p.Ser664Ter)	1	N.A.	Infinity	0.17059	N.A.	Infinity	0.030324	LP
c.3160G>T (p.Glu1054Ter)	1	N.A.	Infinity	0.17059	N.A.	Infinity	0.030324	P
c.2425G>T (p.Glu809Ter)	1	N.A.	Infinity	0.17059	N.A.	Infinity	0.030324	P
***RS1* (NM_000330.4)**								
c.244_245delAC (p.Thr82LeufsTer3)	1	N.A.	Infinity	0.17059	N.A.	Infinity	0.030324	P
***TIMP3* (NM_000362.4)**								
c.428G>A (p.Cys143Tyr)	1	N.A.	Infinity	0.17059	N.A.	Infinity	0.030324	LP

***TTLL5* (NM_015072.5)**								
c.1103A>C (p.Asn368Thr)	1	N.A.	Infinity	0.17059	N.A.	Infinity	0.032849	VUS
c.2212C>T (p.Arg738Ter)	1	N.A.	Infinity	0.17059	0.000218	6.4022	0.16876	P
c.3177_3180delAAAC (p.Asn1060Ter)	2	0.004282	1.7739	0.34065	0.00278	1.096	0.70574	P
***TULP1* (NM_003322.6)**								
c.187G>T (p.Gly63Ter)	1	0.001214	1.321	1	0.000482	2.2857	0.40524	P
***USH2A* (NM_206933.3)**								
c.951delG (p.Tyr318ThrfsTer18)	1	N.A.	Infinity	0.17059	N.A.	Infinity	0.030324	LP
c.2955C>A (p.Cys985Ter)	1	N.A.	Infinity	0.17059	N.A.	Infinity	0.030324	P
c.3665C>T (p.Ala1222Val)	1	0.001318	1.2159	1	0.000655	2.2791	0.3709	LP
c.5104G>A (p.Val1702Met)	1	0.001978	0.8101	1	0.00087	1.8809	0.4259	VUS
c.5603_5613del (p.Phe1868CysfsTer4)	1	N.A.	Infinity	0.17059	N.A.	Infinity	0.030324	P
c.10582A>G (p.Asn3528Asp)	1	N.A.	Infinity	0.17059	N.A.	Infinity	0.032825	LP
c.11288A>G (p.Tyr3763Cys)	3	0.000989	4.8808	0.065971	0.000924	5.0676	0.027848	VUS
c.11712-2A>C	1	N.A.	Infinity	0.17059	0.0000546	29.3981	0.064809	P
c.12503C>A	1	N.A.	Infinity	0.17059	N.A.	Infinity	0.030324	LP

(p.Ser4168Tyr)								
c.13007G>A (p.Cys4336Tyr)	1	N.A.	Infinity	0.17059	0.0000545	29.4751	0.064647	LP
c.13731_13743del (p.Lys4578Ter)	1	N.A.	Infinity	0.17059	N.A.	Infinity	0.030324	P

The odds ratios and *p*-value were calculated by using Fisher's exact test. The population in our cohort is 312, and the corresponding Taiwan and gnomAD East Asian populations were 1,517 and 9,977, respectively. A total of 780 whole-genome sequences of gnomAD East Asian population were used for odds ratios and *p*-value when the variants are within an intron.

Supplementary Table 2 Odds Ratio and *p*-value of Variants Enriched in Our Cohort

Gene variants	Allele count in TIP	Taiwan Biobank			GnomAD East Asia		
		Allele frequency	Odds ratio	*P*-value	Allele frequency	Odds ratio	*P*-value
*ABCA4:*c.1804C>T (p.Arg602Trp)	10	N.A.	Infinity	1.965e-08	0.000272 (5/18364)	59.65065	3.543e-12
*ABCA4:*c.2894A>G (p.Asn965Ser)	4	0.000659 (2/3034)	9.771103	0.009421	0.000435 (8/18394)	16.07433	0.0003413
*CEP290:*c.6798G>A (p.Trp2266Ter)	4	0.002307 (7/3034)	2.788736	0.1021	0.00167 (30/17966)	3.929448	0.0245
*CYP4V2:*c.802-8_810delinsGC	11	N.A.	Infinity	3.307e-09	N.A.	22.30936	1.559e-10
*CYP4V2:*c.1091-2A>G	6	0.000989 (3/3032)	9.793051	0.001274	0.000544 (10/18394)	17.84038	7.364e-06
*EYS:*c.6416G>A (p.Cys2139Tyr)	13	0.002966 (7/3034)	7.145212	1.017e-05	0.00166 (18/10870)	11.99292	3.355e-09
*EYS:*c.7228+1G>A	8	0.001318 (4/3034)	9.828114	0.0001788	0.000337 (3/8904)	33.92573	3.898e-08
*EYS:*c.8107G>T	4	0.002966	2.1679	0.2555	0.00202	3.08142	0.05272

(p.Glu2703Ter)		(7/3034)			(22/10886)		
*RCBTB1:*c.707delA (p.Asn236ThrfsTer11)	6	0.002310 (7/3030)	4.190444	0.01409	0.00146 (22/15060)	5.963604	0.001054
*RLBP1:*c.282delC (p.Phe95SerfsTer24)	5	0.000989 (3/3034)	8.153801	0.005067	0.00125 (23/18390)	6.704771	0.00164
*USH2A:*c.2802T>G (p.Cys934Trp)	6	0.002966 (9/3034)	3.261761	0.03015	0.0025 (46/18376)	3.861062	0.006841

The variants that the allele frequency is more than 0.5% (> 3/624) in our cohort are selected and analyzed. The odds ratios and *p*-value were calculated by using Fisher's exact test. The population in our cohort is 312, and the corresponding Taiwan and gnomAD East Asian populations were 1,517 and 9,977, respectively. A total of 780 whole-genome sequences of gnomAD East Asian population were used for odds ratios and *p*-value when the variants are within an intron.

Supplementary Table 3 212 genes tested in our cohort

Disease category	Genes
Bardet-Biedl syndrome, AR	*ARL6, BBIP1, BBS1, BBS2, BBS4, BBS5, BBS7, BBS9, BBS10, BBS12, C8orf37, CEP290, IFT172, IFT27, INPP5E, KCNJ13, LZTFL1, MKKS, NPHP1, SDCCAG8, TRIM32, TTC8*
Chorioretinal atrophy or degeneration, AD	*PRDM13, RGR*
Cone or cone-rod dystrophy, AD	*AIPL1, CRX, GUCA1A, GUCY2D, PITPNM3, PROM1, PRPH2, RIMS1, SEMA4A, UNC119*
Cone or cone-rod dystrophy, AR	*ABCA4, ADAM9, ATF6, C21orf2, C8orf37, CACNA2D4, CDHR1, CERKL, CNGA3, CNGB3, CNNM4, GNAT2, KCNV2, PDE6C, PDE6H, POC1B, RAB28, RAX2, RDH5, RPGRIP1, TTLL5*
Cone or cone-rod dystrophy, XL	*CACNA1F, RPGR*
Congenital stationary night blindness, AD	*GNAT1, PDE6B, RHO*
Congenital stationary night blindness, AR	*CABP4, GNAT1, GNB3, GPR179, GRK1, GRM6, LRIT3, RDH5, SAG, SLC24A1, TRPM1*
Congenital stationary night blindness, XL	*CACNA1F, NYX*
Deafness alone or syndromic, AD	*WFS1*
Deafness alone or syndromic, AR	*CDH23, CIB2, DFNB31, MYO7A, PCDH15, USH1C*
Leber congenital amaurosis, AD	*CRX, IMPDH1, OTX2*
Leber congenital amaurosis, AR	*AIPL1, CABP4, CEP290, CRB1, CRX, DTHD1, GDF6, GUCY2D, IQCB1, KCNJ13, LCA5, LRAT, NMNAT1, PRPH2, RD3, RDH12, RPE65, RPGRIP1, SPATA7, TULP1*
Macular degeneration, AD	*BEST1, C1QTNF5, ELOVL4, FSCN2, GUCA1B, HMCN1, IMPG1, OTX2, PRDM13, PROM1, PRPH2, RP1L1, TIMP3*
Macular degeneration, AR	*ABCA4, DRAM2, IMPG1*
Macular degeneration, XL	*RPGR*
Age-related macular degeneration (AMD)	*ABCA4 FBLN5, HMCN1, RAX2*
Retinitis pigmentosa, AD	*BEST1, CA4, CRX, FSCN2, GUCA1B, HK1, IMPDH1, IMPG1, KLHL7, NR2E3, NRL, PRPF3, PRPF4, PRPF6, PRPF8, PRPF31, PRPH2, RDH12, RHO, ROM1, RP1, RP9, RPE65, SAG, SEMA4A, SNRNP200, TOPORS*

Retinitis pigmentosa, AR	*ABCA4, AGBL5, ARL6, ARL2BP, BBS1, BBS2, BEST1, C2orf71, C8orf37, CERKL, CLRN1, CNGA1, CNGB1, CRB1, CYP4V2, DHDDS, DHX38, EMC1, EYS, FAM161A, HGSNAT, IDH3B, IFT172, IMPG2, KIAA1549, KIZ, LRAT, MAK, MERTK, MVK, NEK2, NEUROD1, NR2E3, NRL, PDE6A, PDE6B, PDE6G, PRCD, PROM1, RBP3, RGR, RHO, RLBP1, RP1, RP1L1, RPE65, SAG, SAMD11, SLC7A14, SPATA7, TTC8, TULP1, USH2A, ZNF408, ZNF513*
Retinitis pigmentosa, XL	*OFD1, RP2, RPGR*
Syndromic/systemic diseases with retinopathy, AD	*ABCC6, ATXN7, COL11A1, COL2A1, JAG1, KCNJ13*
Syndromic/systemic diseases with retinopathy, AR	*ABCC6, ABHD12, ACBD5, ADAMTS18, AHI1, ALMS1, CC2D2A, CEP290, COL9A1, CSPP1, ELOVL4, FLVCR1, GNPTG, HARS, HGSNAT, INPP5E, INVS, IQCB1, LAMA1, LRP5, NPHP1, NPHP3, NPHP4, PANK2, PCYT1A, PEX1, PEX2, PEX7, PHYH, PNPLA6, POC1B, PRPS1, RDH11, RPGRIP1L, SDCCAG8, TMEM216, TMEM237, TTPA, TUB, WDPCP, WFS1, ZNF423*
Syndromic/systemic diseases with retinopathy, XL	*OFD1,*
Usher syndrome, AR	*ABHD12, CDH23, CEP250, CIB2, CLRN1, DFNB31, HARS, MYO7A, PCDH15, USH1C, USH1G, USH2A*
Other retinopathy, AD	*BEST1, CRB1, FZD4, ITM2B, LRP5, MAPKAPK3, MIR204, OPN1SW, RCBTB1, TSPAN12, ZNF408*
Other retinopathy, AR	*ASRGL1, BEST1, CDH3, CNGA3, CNGB3, CNNM4, CYP4V2, LRP5, MFRP, MVK, NR2E3, OAT, PROM1, RCBTB1, RLBP1*
Other retinopathy, XL	*CACNA1F, CHM, NDP, OPN1LW, OPN1MW, PGK1*
Other putative genes	*ADGRA3, ARL13B, CLRN3, COL11A2, GPR143, OR2W3, PEX26, TMEM67, RS1*
Macular degeneration, AD	*BEST1, C1QTNF5, ELOVL4, FSCN2, GUCA1B, HMCN1, IMPG1, OTX2, PRDM13, PROM1, PRPH2, RP1L1, TIMP3*

AD, autosomal dominant; AR, autosomal recessive; XL, X-linked. Note: The untranslated regions (5'UTR and 3'UTR), exons, and introns were captured for some genes (*USH2A, OFD1, ABCA4, PRPF31 CEP290, RPGR, GUCY2D, KCNV2, CNGB3, CNGA3, PRPF4, MYO7A, RPGRIP1, RDH12, AIPL1, CNGB1, NRL, SPATA7, FAM161A, RPE65, PDE6A, PDE6B, BBS10,* and *BBS1*).

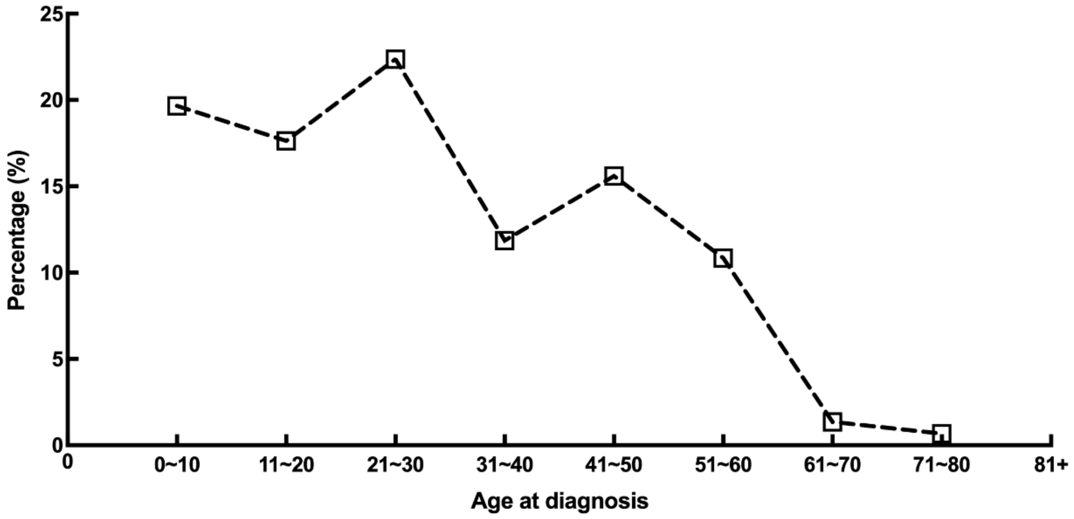

Supplementary Figure 1. The distribution of age at diagnosis in our cohort.

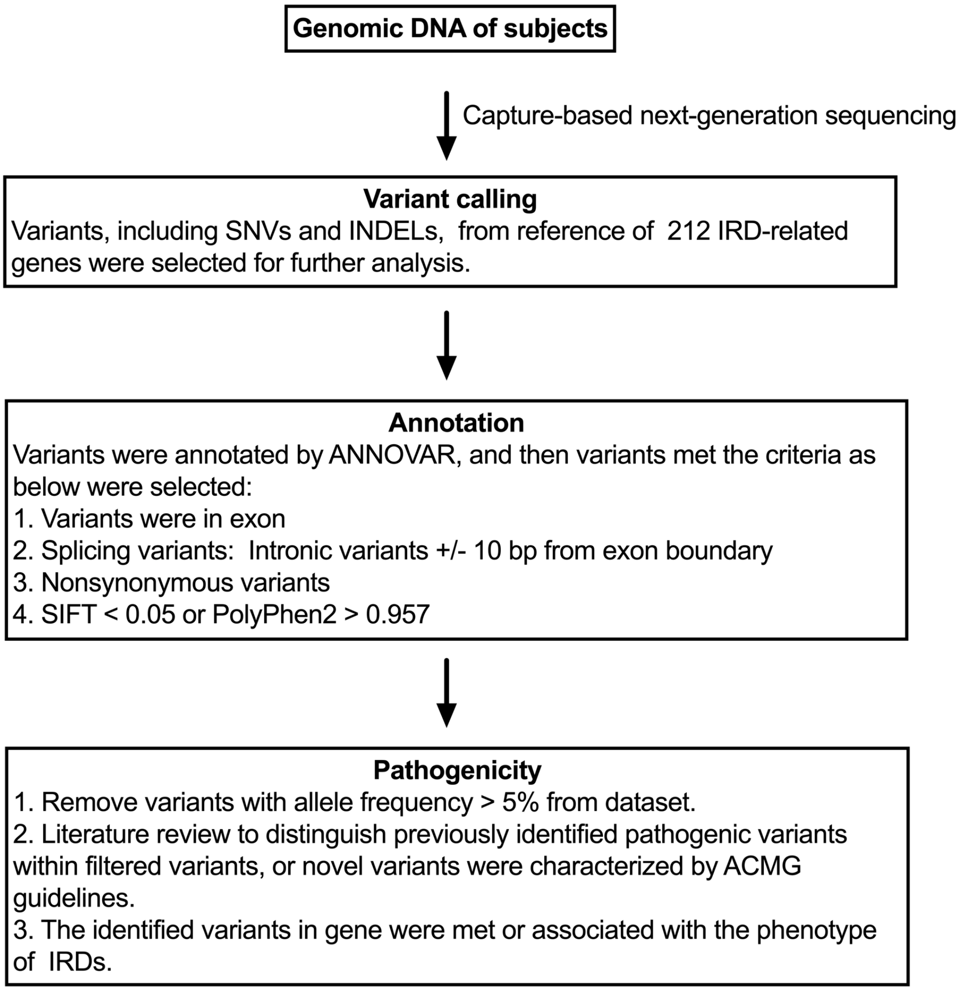

Supplementary Figure 2. Data-analysis pipeline. SNVs = single nucleotide variants; INDELs = insertions/deletions
