# Supplementary material for: Genetic characteristics and epidemiology of inherited retinal degeneration in Taiwan
Source: NPJ Genom Med. 2021 Feb 19;6:16. doi: 10.1038/s41525-021-00180-1 (PMC7896090; doi:10.1038/s41525-021-00180-1)
Supplement: Supplementary file 3 — Reporting Summary [file 41525_2021_180_MOESM3_ESM.pdf]

nature research

Corresponding author(s): FRH, PLC

Last updated by author(s): Nov 29, 2020

Reporting Summary

Nature Research wishes to improve the reproducibility of the work that we publish. This form provides structure for consistency and transparency in reporting. For further information on Nature Research policies, see our Editorial Policies and the Editorial Policy Checklist.

Statistics

For all statistical analyses, confirm that the following items are present in the figure legend, table legend, main text, or Methods section.

n/a	Confirmed	
☐	☒	The exact sample size (n) for each experimental group/condition, given as a discrete number and unit of measurement
☐	☒	A statement on whether measurements were taken from distinct samples or whether the same sample was measured repeatedly
☐	☒	The statistical test(s) used AND whether they are one- or two-sided<br>*Only common tests should be described solely by name; describe more complex techniques in the Methods section.*
☐	☒	A description of all covariates tested
☐	☒	A description of any assumptions or corrections, such as tests of normality and adjustment for multiple comparisons
☐	☒	A full description of the statistical parameters including central tendency (e.g. means) or other basic estimates (e.g. regression coefficient) AND variation (e.g. standard deviation) or associated estimates of uncertainty (e.g. confidence intervals)
☐	☒	For null hypothesis testing, the test statistic (e.g. F, t, r) with confidence intervals, effect sizes, degrees of freedom and P value noted<br>*Give P values as exact values whenever suitable.*
☒	☐	For Bayesian analysis, information on the choice of priors and Markov chain Monte Carlo settings
☒	☐	For hierarchical and complex designs, identification of the appropriate level for tests and full reporting of outcomes
☒	☐	Estimates of effect sizes (e.g. Cohen's d, Pearson's r), indicating how they were calculated

Our web collection on statistics for biologists contains articles on many of the points above.

Software and code

Policy information about availability of computer code

Data collection	No software was used
Data analysis	No software was used

For manuscripts utilizing custom algorithms or software that are central to the research but not yet described in published literature, software must be made available to editors and reviewers. We strongly encourage code deposition in a community repository (e.g. GitHub). See the Nature Research guidelines for submitting code & software for further information.

Data

Policy information about availability of data

All manuscripts must include a data availability statement. This statement should provide the following information, where applicable:

- Accession codes, unique identifiers, or web links for publicly available datasets
- A list of figures that have associated raw data
- A description of any restrictions on data availability

The sequencing raw data (FASTQ files) analyzed during the current study available in the NCBI Sequence Read Archive (SRA) (PRJNA681323).

Field-specific reporting

Please select the one below that is the best fit for your research. If you are not sure, read the appropriate sections before making your selection.

☒ Life sciences ☐ Behavioural & social sciences ☐ Ecological, evolutionary & environmental sciences

For a reference copy of the document with all sections, see nature.com/documents/nr-reporting-summary-flat.pdf

Life sciences study design

All studies must disclose on these points even when the disclosure is negative.

Sample size	312 probands with IRDs
Data exclusions	All probands were included because they are clinically diagnosed as IRDs.
Replication	Approximately two-thirds of the patients were diagnosed with retinitis pigmentosa (RP, n = 205, 65.7%), the most prevalent phenotype in our cohort, followed by macular dystrophy (MD, n = 39, 12.5%), cone dystrophy/cone-rod dystrophy (CD/CRD, n = 19, 6.1%), Leber congenital amaurosis (LCA, n = 16, 5.1%), Bietti's crystalline dystrophy (BCD, n = 12, 3.8%), occult macular dystrophy (OMD, n = 5, 1.6%), retinoschisis (RS, n = 4, 1.3%), choroideremia (n = 2, 0.6%), familial exudative vitreoretinopathy (FEVR, n = 2, 0.6%), Alstrom syndrome (n = 2, 0.6%), and others (n = 6, 1.9%)
Randomization	The genomic DNA of all probands are perfomed by capture-based NGS, and the diagnosis are refined after genetic diagnosis.
Blinding	The genomic DNA of all probands are perfomed by capture-based NGS, and the diagnosis are refined after genetic diagnosis.

Reporting for specific materials, systems and methods

We require information from authors about some types of materials, experimental systems and methods used in many studies. Here, indicate whether each material, system or method listed is relevant to your study. If you are not sure if a list item applies to your research, read the appropriate section before selecting a response.

Materials & experimental systems

n/a	Involved in the study	
☒	☐	Antibodies
☒	☐	Eukaryotic cell lines
☒	☐	Palaeontology and archaeology
☒	☐	Animals and other organisms
☐	☒	Human research participants
☒	☐	Clinical data
☐	☒	Dual use research of concern

Methods

n/a	Involved in the study	
☒	☐	ChIP-seq
☒	☐	Flow cytometry
☒	☐	MRI-based neuroimaging

Human research participants

Policy information about studies involving human research participants

Population characteristics	From July 2015 to June 2019, 312 families with IRDs were identified and recruited consecutively into our TIP project. During a 48-month period, 587 patients of IRDs in the 312 families were approached and the first patient of each family recruited into our project was regarded as the "proband." This statistical analysis in this study is based on the proband of each affected family to minimize the potential bias in genetic epidemiology. For every subject recruited, detailed ophthalmological examinations were performed, including measurement of best-corrected visual acuity, electroretinograms, color fundus photography, optical coherence tomography, and fundus autofluorescence imaging to confirm the clinical diagnosis of IRDs. A detailed medical history, including pedigree, was obtained for every proband.
Recruitment	Patients with IRD were recruited either from our out-patient clinic or from patients referred by other medical centers in Taiwan to the Department of Ophthalmology, National Taiwan University Hospital (NTUH).
Ethics oversight	The present study was conducted at the Department of Ophthalmology, National Taiwan University Hospital (NTUH), and was approved by the Research Ethics Committee of the National Taiwan University Hospital (IRB NO.: 201408082RINC).

Note that full information on the approval of the study protocol must also be provided in the manuscript.

Dual use research of concern

Policy information about dual use research of concern

Hazards

Could the accidental, deliberate or reckless misuse of agents or technologies generated in the work, or the application of information presented in the manuscript, pose a threat to:

No	Yes	
☒	☐	Public health
☒	☐	National security
☒	☐	Crops and/or livestock
☒	☐	Ecosystems
☒	☐	Any other significant area

Experiments of concern

Does the work involve any of these experiments of concern:

No	Yes	
☒	☐	Demonstrate how to render a vaccine ineffective
☒	☐	Confer resistance to therapeutically useful antibiotics or antiviral agents
☒	☐	Enhance the virulence of a pathogen or render a nonpathogen virulent
☒	☐	Increase transmissibility of a pathogen
☒	☐	Alter the host range of a pathogen
☒	☐	Enable evasion of diagnostic/detection modalities
☒	☐	Enable the weaponization of a biological agent or toxin
☒	☐	Any other potentially harmful combination of experiments and agents
